# Enzyme Biosensors for Biomedical Applications: Strategies for Safeguarding Analytical Performances in Biological Fluids

**DOI:** 10.3390/s16060780

**Published:** 2016-05-30

**Authors:** Gaia Rocchitta, Angela Spanu, Sergio Babudieri, Gavinella Latte, Giordano Madeddu, Grazia Galleri, Susanna Nuvoli, Paola Bagella, Maria Ilaria Demartis, Vito Fiore, Roberto Manetti, Pier Andrea Serra

**Affiliations:** Department of Clinical and Experimental Medicine, Medical School, University of Sassari, Viale S. Pietro 43/b, Sassari 07100, Italy; angela.spanu@email.it (A.S.); babuder@uniss.it (S.B.); avi@live.it (G.L.); giordano@uniss.it (G.M.); graziagalleri@gmail.com (G.G.); snuvoli@uniss.it (S.N.); paola.bagella@tiscali.it (P.B.); mardemartis@uniss.it (M.I.D.); vitofiore30010516@gmail.com (V.F.); rmanetti@uniss.it (R.M.); paserra@uniss.it (P.A.S.)

**Keywords:** biosensor, biomedical applications, amperometry, biological fluids, interferents

## Abstract

Enzyme-based chemical biosensors are based on biological recognition. In order to operate, the enzymes must be available to catalyze a specific biochemical reaction and be stable under the normal operating conditions of the biosensor. Design of biosensors is based on knowledge about the target analyte, as well as the complexity of the matrix in which the analyte has to be quantified. This article reviews the problems resulting from the interaction of enzyme-based amperometric biosensors with complex biological matrices containing the target analyte(s). One of the most challenging disadvantages of amperometric enzyme-based biosensor detection is signal reduction from fouling agents and interference from chemicals present in the sample matrix. This article, therefore, investigates the principles of functioning of enzymatic biosensors, their analytical performance over time and the strategies used to optimize their performance. Moreover, the composition of biological fluids as a function of their interaction with biosensing will be presented.

## 1. Introduction

In recent decades, biosensing has proven to be an innovative technique in several fields, from the environment, to biomedical applications. Modern biosensors can be miniaturized, mass produced and easily transported. Biosensors can also measure analytes in real-time, which is extremely useful for monitoring rapid changes in biological fluids.

A biosensor is universally defined as “a self-contained analytical device that combines a biological component with a physicochemical device for the detection of an analyte of biological importance” [1]. It consists of a biological recognition element which is able to specifically interact with a target molecule and a transducer able to convert this interaction into a measurable signal.

Chemical biosensors are based on the presence of a biological element, which is specific for the analyte, and stable under normal conditions of use and storage [2]. Numerous recognition elements have been used in biosensors, such as receptors, nucleic acids, whole cells, antibodies and different class of enzymes.

Biosensors are normally classified according to the transduction method they use. In biosensors, the transducer converts a wide array of chemical, physical or biological reactions into an electrical signal [3]. On this basis, optical, calorimetric or acoustic biosensors have been built and characterized, but the most widely used biosensors rely on the electrochemical proprieties of transducers and analytes. Electrochemical biosensors have been studied since the early 1960s when the first glucose oxidase biosensor was developed [4]. Electrochemical biosensors can be impedimetric, potentiometric or amperometric biosensors, where the biochemical signal is transduced into a quantifiable amperometric signal [5]. Enzyme-based amperometric biosensors, in which the production of a current is monitored when a fixed potential is applied between two electrodes, have been widely studied over the last few decades as they are easy to miniaturize, robust and can operate with small sample volumes of rather complex matrices [6,7].

Designing biosensors requires consideration of both the target analyte and the complexity of the matrix in which the analyte will be measured. Electrochemical measurements depend strongly on the working electrode material [8]. Since the end of 1980s, research has focused on the development of amperometric biosensors based on carbon paste electrodes [9]. Carbon still represents one of the most widely-used material for biosensing in electrocatalysis and electroanalysis, exploiting the favourable chemical-physical proprieties of carbon nanotubes or graphene, as well as desirable catalytic proprieties (high surface area, good biocompatibility, chemical stability and signal reproducibility) [10]. In addition, metals, such as gold, platinum or palladium, have been used as transducers in electrochemical biosensors as electron transfer is easy and hydrogen peroxide generated by first generation oxidase based biosensors is efficiently electro-oxidized to generate a signal [11,12].

The characteristics noted above make biosensors useful for biomedical application, research and, even in some cases, for diagnosis. Biomedical biosensors are cost-effective, easy to use, fast and can use wireless detection [13]. Castillo *et al.* reported in 2004, that enzyme-based amperometric biosensors could be used in the following ways [14]:

- As “off line” devices — biological samples are collected and target analytes are measured using biosensor-based analytical equipment. For example, commercial devices are available for measuring blood glucose.
- As “*in vivo*” sensors — biosensors are implanted and continuously detect extracellular changes in the concentrations of the analyte of interest. The invasiveness of such implantable devices limits their use mainly to preclinical research in animal models.
- As “on-line” device — biosensors are integrated with a sampling device implanted in the body or biological material. For instance, microdialysis probes can be implanted and connected to a flow through detector incorporating a biosensor element.

Amperometric enzyme-based biosensors are subject to interference from chemicals present in the sample matrix. Interference is especially problematic in biological samples in particular, as there are often electrochemical interferences in the sample matrix [15], as well as small molecule metabolites, proteins, macromolecules and cells. Complex biochemical reactions occur naturally in these fluids (for example, blood clotting) [16]. Some pathological conditions, such as inflammation or tumors, could modify some fluid parameters chemical composition or pH, influencing the activities of the enzyme and, consequently, the biosensor performances. Matrix interference can often be overcome by pretreatments, such as extraction, pre-concentration, filtration and derivatization [17]. However, the direct use of biosensors in a matrix “as is” is preferable because it avoids the sample preparation procedures.

In this review, the principles of enzymatic biosensors function will be discussed in Section 2, while their analytical performance over time and strategies used to optimize performance will be discussed in Section 3. The composition of biological fluids as a function of their interaction with biosensors will be presented in the last section.

## 2. Amperometric Enzyme Biosensors

Amperometric enzyme biosensors are commonly divided into three main classes, or generations, depending on the electron transfer method used for the measurement of the biochemical reaction [18] or the degree of separation of the biosensor components (transducer, enzyme, mediators and cofactors) [19]. In all cases, the presence of an enzyme is required and therefore sensor performance relies on different parameters, such as working pH and temperature.

### 2.1. First Generation Biosensors

**Figure 1:**
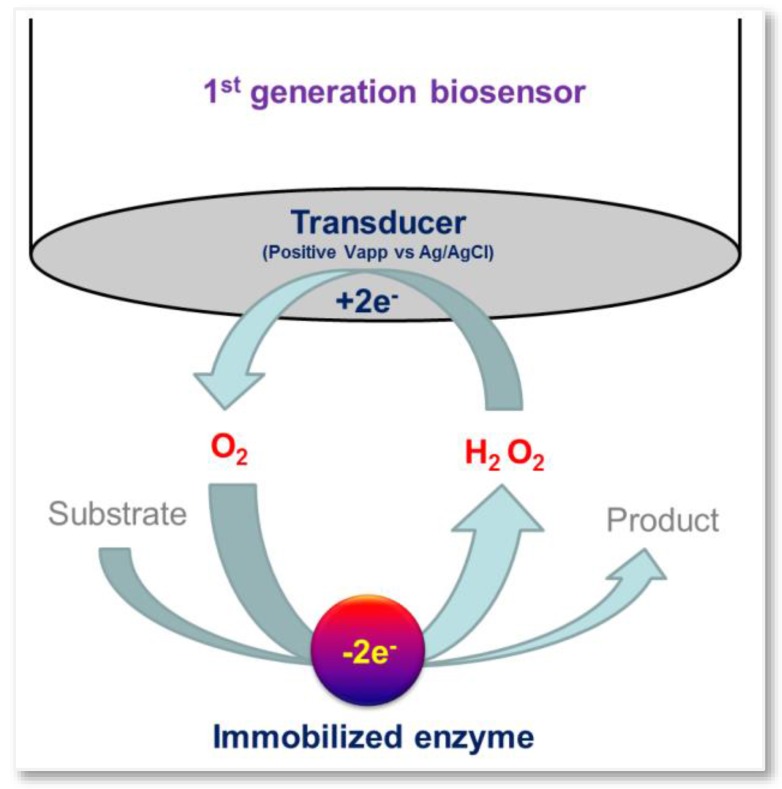
Schematic representation of a first generation biosensor.

First generation biosensors measure the concentration of analytes and/or products of enzymatic reactions that diffuse to the transducer surface and generate an electrical response. They are also called mediatorless amperometric biosensors [20] (Figure 1).

In this class of biosensors, the enzyme is immobilized on a transducer surface and its capability to transform a substrate in an electroactive, measurable byproduct is exploited. Such biosensors rely on enzymes that belong to two main categories: oxidases (Table 1) and dehydrogenases (Table 2). Oxidases and dehydrogenases require coenzymes during catalysis (for example, NAD^+^, NADP^+^, NADH, NADPH, ATP FAD, FADH), which need to be regenerated to allow the enzyme to catalyze subsequent reactions. For example, when oxidase enzymes are involved, the following reactions occur (Equations (1) and (2):

S+E-FAD **→** E-FAD-S **→** E-FADH_2_+P (1)

E-FADH_2_+O_2_**→** E-FAD +H_2_O_2_ (2)

In oxidase enzymes, the most common co-factor is flavin adenine dinucleotide (FAD), which is not covalently bonded to the enzyme [21]. Oxidase-based biosensors can either monitor the production of hydrogen peroxide (H_2_O_2_) by applying a fixed anodic potential (+0.7 V *vs.* Ag/AgCl) or oxygen (O_2_) consumption by applying a fixed cathodic potential (−0.7 V *vs.* Ag/AgCl). An alternative approach, which is extensively used in the detection of hydrogen peroxide, is the introduction of a peroxidase in the biosensor design, which allows for the detection of H_2_O_2_ by applying a low reducing potential [22].

Oxidase enzymes need molecular oxygen as a second substrate so the oxidase-based biosensors are oxygen dependent. First generation biosensors that use oxygen as an electron acceptor are thus subject to errors arising from changing or low concentration of dissolved oxygen impacting on sensor response and reducing linearity [23]. This oxygen dependence limits the applicability of first generation amperometric biosensors in biological systems—for example, they are not suitable for use under ischemic conditions [24].

First generation biosensors based on the use of dehydrogenase enzymes [52], give rise to the following reaction scheme:

S + E + NAD^+^**→** S-E-NAD^+^**→** E + P + NADH + H^+^ (3)

NADH **→** NAD^+^ + H^+^ + 2e^−^ (4)

For this type of biosensor, the NADH concentration is directly proportional to the concentration of the monitored analyte. NADH must be present in the matrix in order to give rise to a signal [53], which becomes problematic for implantable biosensors. Accurate detection of the target analyte requires adequate cofactor concentrations.

First generation biosensors have proven to be highly sensitive and are characterized by a very low response-times, typically around one second [35]. However, first generation biosensors often require electrode pretreatment in order to generate a reproducible surface and sensor response, and corrections for matrix effects related to interference are often necessary [20]. In addition, prolonged use of amperometric biosensors, especially in complex biological matrices or undiluted samples, often results in fouling of the surface of the transducers [67], affecting the biosensor response.

### 2.2. Second Generation Biosensors

**Figure 2:**
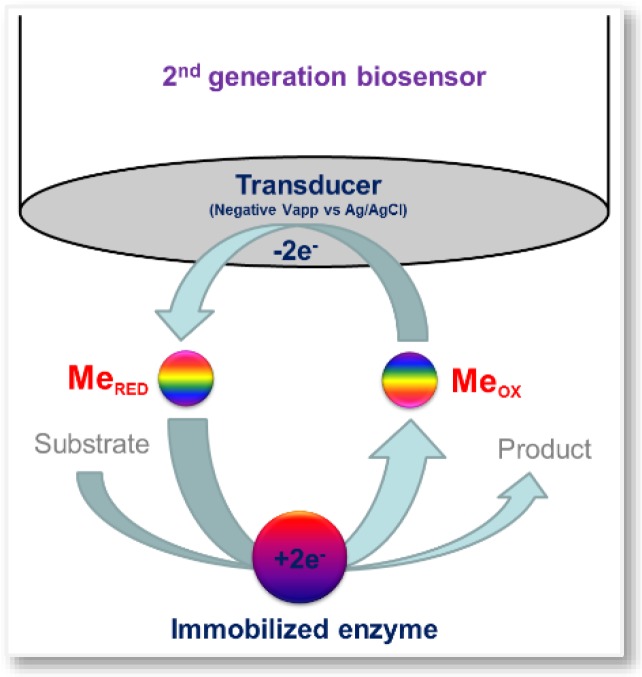
Scheme of a second generation biosensor; Me_OX_: oxidized mediator; Me_RED_: reduced mediator.

Second generation biosensors, also called mediator amperometric biosensors (Figure 2), exploit mediators, as oxidizing agents, to act as electron carriers [68]. This approach makes it possible to work at low potentials, avoiding O_2_ dependence and the impact of interfering molecules. The most common and well-known mediators are ferricyanide and ferrocene, although, methylene blue, phenazines, methyl violet, alizarin yellow, Prussian blue, thionin, azure A and C, toluidine blue and inorganic redox ions are also widely used [69]. Further improvements are obtained by replacing oxygen with an electron acceptor capable of carrying electrons from the redox center of the enzyme (E) to the electrode.

The reaction takes place according to the following scheme:

S + E(ox) **→** P + E(red) (5)

E(red) + 2M_OX_ **→** E(ox) + 2M_RED_ + 2H^+^ (6)

2M_RED_ **→** 2M_OX_ + 2e^-^ (7)

where M_OX_ and M_RED_ are the oxidized and reduced forms of the mediator and M_RED_ is oxidized at the electrode surface, giving a current signal proportional to the detected analyte concentration [23]. 

Mediators can be added to the sample or immobilized on the electrode surface. For immobilized mediators, it is extremely important that mediator is entrapped close to the enzyme and that if the mediator is lost, the time course of loss is known in order to characterize the biosensor performance over time. Suitable mediators are stable during the reaction under working conditions and are not involved in the electron transfer. Moreover, the mediator should have a lower redox potential than other electroactive compounds in the sample [69]. Second generation biosensors are less commonly used than first generation biosensors as they general have low stability due to the immobilized mediators. 

### 2.3. Third Generation Biosensors

**Figure 3:**
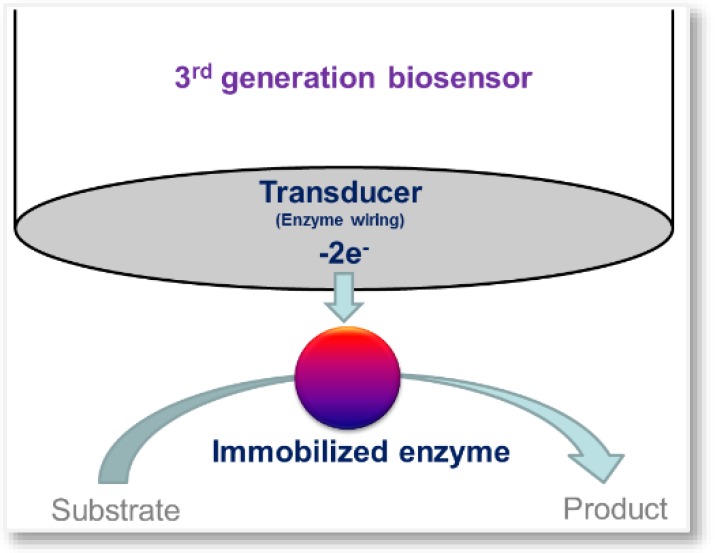
Simplified drawing of a third generation biosensor.

Third generation biosensors rely on bioelectrocatalysis [20], where there is a direct electron transfer between enzyme and electrode (Figure 3). 

A third generation biosensor consists of three elements: the enzyme as bio-recognition element, the redox polymer (or the nano-scale wiring element) to ensure the signal propagation and the electrode as the entrapping surface [67]. Using a redox polymer to “wire” the redox center of the sensing enzyme to the electrode surface improves performance. Third-generation biosensors are still being developed and are not commonly used for analysis. However, developments in polymer science and nanotechnology make third generation biosensors promising, as the sensors are likely to have very short response times and be relatively independent of oxygen/cofactor concentrations.

### 2.4. pH and Temperature Dependence

**Figure 4:**
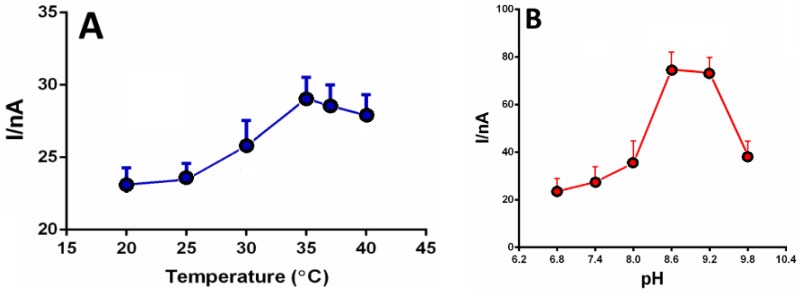
Temperature (**A**) and pH (**B**) dependence on alcohol oxidase from *Hansenula polymorfa*, immobilized on platinum surface [34].

The catalytic activity of enzymes is strongly dependent on pH and temperature. These differences can be attributed to the substrate, oxygen, and the enzyme itself [70]. For example, in previous studies [34], we demonstrated that the optimal working temperature of alcohol oxidase immobilized on a platinum electrode was between 35 °C and 40 °C over a pH range of 8.6 to 9.2 (Figure 4).

Reaction rates increase exponentially with temperature, up to 50 °C beyond which irreversible thermal denaturation of the protein occurs for most enzymes [71]. pH dependence varies by enzyme, matrix composition and temperature [72]. At the optimum pH and temperature, biosensor responses are reproducible and greatest sensitivity is achieved. For instance, the working pH range for laccase is between 3 and 7, while for enzymes derived from plants pH 9 is optimal [71]. Importantly, as long as pH and temperature dependences are accounted for, biosensors can be accurate even if operating outside their optimum conditions.

### 2.5. Enzyme as Label Element Instead of Recognition Element

**Figure 5:**
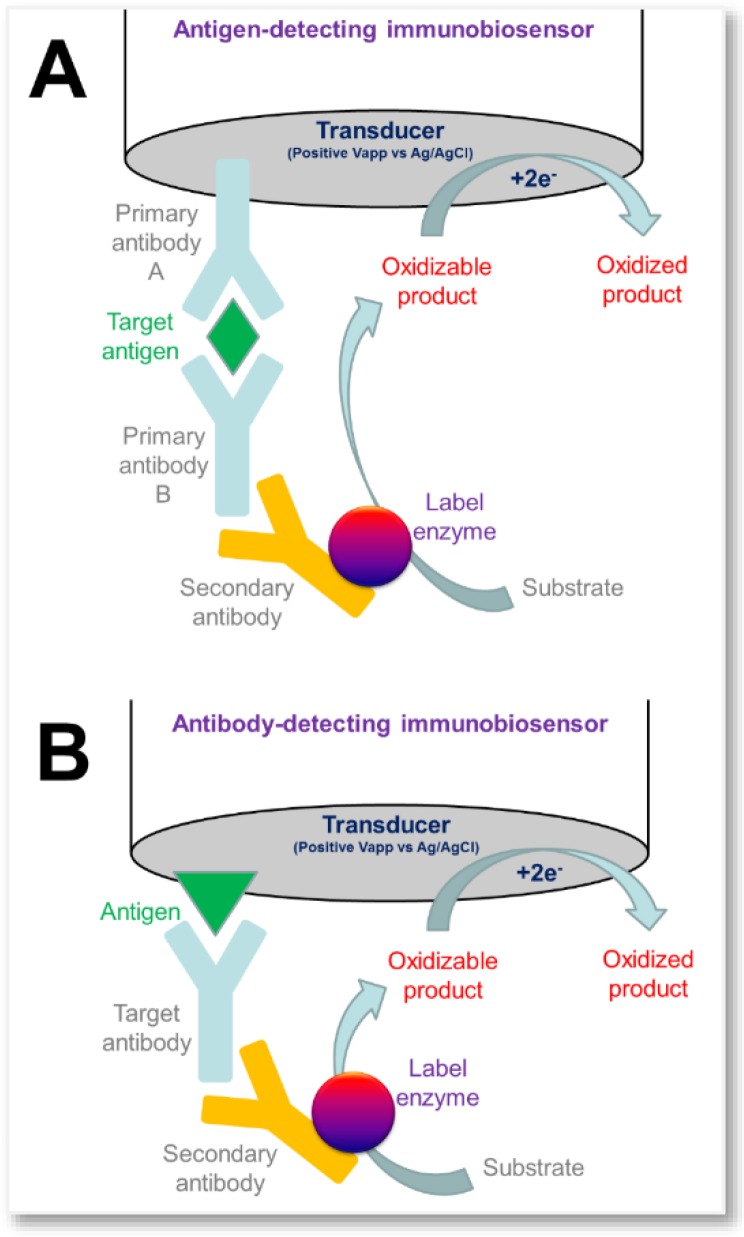
Examples of immune-biosensors that use an enzyme as label element for the indirect detection of a target antigen (panel A) or a target antibody (panel B). These bio-electrochemical reactions are carried-out in a controlled environment without matrix-related interferences.

Amperometric biosensors normally exploit enzymes as a bio-recognition element. However, biosensors can also be developed using an alternative approach, similar to ELISA assays, where enzymes are used to extract analyte from the sample matrix, followed by complexation with an antibody labeled with a secondary enzyme [73]. One example of this approach is a screen-printed immunosensor for the quick measurement of α-1-fetoprotein (AFP) in human serum (Figure 5), where horseradish peroxidase (HRP)-labeled AFP antibody is entrapped in a chitosan membrane [74]. Aptamer-based biosensors also operate on the same principles. For example, glucose dehydrogenase has been used as labelling enzyme to monitor thrombin using a glucose dehydrogenase (GDH)-labeled anti-thrombin aptamer, where a second aptamer is immobilized on the electrode surface [75]. Thrombin has also been detected by a new analytical approach using aptamer-functionalized magnetic beads [76].

Nowadays, the development of immune-based biosensors is moving towards removal of enzymes from the design (unlabeled immune-biosensors) in order to decrease the complexity of manufacturing. In this case, the interaction between the biological element (for example, antibody or aptamer) and its target is quantified by measuring the diffusibility of an electroactive compound (for example, ferrocene) to the transducer surface.

## 3. Enzyme Biosensor Analytical Performance over Time

### 3.1. Biofouling, Electrode Passivation, Enzyme Inactivation and Loss

There are two categories of biosensor failure: “component-based failure” due to lead detachment or electrical shorts and “biocompatibility-based failure” due to the membrane biofouling, electrode passivation or membrane biodegradation. Membrane biofouling is a common cause for biosensor failure in *in vivo* applications. With biofouling, proteins, cellular debris or living cells adhere or adsorb to the outer biosensor surface, impeding analyte diffusion at the biosensor surface, ultimately leading to a decrease in sensor response. Electrode passivation is another type of “bio-compatibility-based failure”, different from membrane biofouling, that occurs when small molecules are transformed to adherent substances at the electrode surface, reducing its active area [77].

There are many chemicals in biological matrices that may cause biofouling and passivation. Phenolic compounds, for example, produce a polymeric film on transducer surfaces when a suitable voltage is applied, decreasing biosensor signals [78,79]. Proteins, as well as other macromolecules present in biological fluids, likely contribute to biofouling by adsorption [80]. Small molecular weight proteins are also likely to be able to block sensor membranes, reducing analyte diffusion.

Adsorption of macromolecules, small surface active compounds and electrode reaction products are responsible of several effects: inhibit electrocatalysis, hinder diffusion and affect the partitioning of the analyte [81]. Adsorbtion can be assessed by using a.c. impedance measurements [82] or cyclic voltammetry (CV) by studying the double-layer region of the voltammogram [81]. The absorption of proteins (affecting the width of CV peaks or the charge transfer resistance of a.c impedance) reduces the biosensor performance by occupying surface sites, important for enzymes catalytic activity. Also diffusion can be studied with cyclic voltammetry by using a reversible electron transfer couple such as hexaammineruthenium (III): a decreased diffusion coefficient in the vicinity of the electrode corresponds to the decrease of the current intensity of redox peaks [81].

Elements active in the biosensor itself may also contribute to passivation. For example, in dehydrogenase-based biosensors, the direct oxidation of NADH often results in electrode fouling by two different mechanisms. Firstly, NAD radicals react directly with oxide moieties on the electrode surface. Secondly, NAD^+^, can adsorb onto the electrode surface. Both of these processes are irreversible and produce a gradual passivation of the electrode during continued biosensor operation [83].

Wisniewski *et al*. reported that biofouling is still a significant challenge for implantable biosensors, although there are strategies to eliminate, or at least reduce, the phenomenon. Electrode fouling can be prevented by selecting an operating voltage that oxidizes or reduces the analyte of interest, but does not promote the formation of passivating polymeric films [84]. Electropolymerised films have also been shown to prevent electrode fouling [85].

The lifetime of enzymatic biosensors is also related to the loss of enzyme activity over time. Loss of activity for immobilized enzymes is principally due to denaturation and deactivation of the protein, which ultimately reduces the life of the sensor [86]. There are two types of stability that are relevant in biosensor development: shelf-life and operational stability. Storage conditions of the biosensor after manufacture but before use can affect the retention of biosensor enzyme activity. Operational stability is related to the ability of the biosensor enzymes to maintain their activity during use, and impacts on the operating lifetime and reproducibility of a biosensor [87]. Enzymes can be inactivated in a number of ways depending on the operational conditions, and it is likely that several deactivation mechanisms may occur at the same time. Mechanisms of enzyme activity loss involve modifications in the three-dimensional structure of the protein that alter the native protein conformation and affect enzyme active sites [88]. Generally, dried enzymes are more stable than solubilized solutions and immobilized enzymes tend to show better stability than pure enzymes [87]. Commonly, stabilization of biosensor enzymes is achieved by a combination of different methods that prevent or minimise degradation. 

Effective immobilization approaches can preserve enzymatic activity and improve biosensor stability [89]. However, the immobilization process can affect enzyme activity and stability, some immobilization techniques yield random distribution or poor orientation of enzymes resulting in partial or total loss of activity due to denaturation of the enzyme or blocking analyte access to the catalytic site [89,90]. Moreover, enzyme denaturation can occur during immobilization, reducing catalytic activity or altering the properties of the biocatalyst [91].

### 3.2. Strategies to Increase Enzyme Selectivity, Specificity and Lifetime

Different strategies have been used to improve the performance of biosensors. In addition to improving biosensor stability, as outlined above, immobilization can also be used to improve sensitivity, selectivity, response time, as well as operational and storage stability and reproducibility [89]. Biosensor sensitivity, selectivity and reproducibility. For example, isolation of enzymes from the biological matrix by depositing a nanofiltration membrane has been shown to increase catalytic activity by 20-fold as a result of poor penetration of macromolecules [92]. Common immobilization strategies include entrapment, cross-linking, adsorption, covalent or affinity linking [93,94,95]. Polyurethane, cellulose acetate and porous silicone polymeric membranes have been used to immobilize enzymes and reject electroactive interferents while still allowing analytes to reach the catalytic sites [96]. Different immobilization methods have advantages and disadvantages, so selection of the optimum method is very important [89].

#### 3.2.1. Adsorption

Adsorption represents the easiest way of depositing enzymes on the electrode surface [72]. Enzymes are deposited by dipping the electrodes in an enzyme solution for a set time period [97] or by adsorption and dip-evaporation, sometimes followed by exposure to glutaraldehyde [98] or other agents as glyoxal or hexamethylenediamine [89], in order to crosslink the enzymes. While adsorption approaches are simple, the enzymes are not deposited in an ordered fashion.

#### 3.2.2. Sol–Gel Process

The use of sol-gels for enzyme immobilization in biosensors started in the 1990s [99,100,101,102]. Sol–gel formation is an easy immobilization method that results in a stable material where enzyme activity is preserved and biosensor sensitivity is enhanced. Sol-gel immobilization is conducted at low temperature so it is compatible with biomolecules. Currently, there are sol-gel methods for generating nanomaterials that look promising for biosensor applications [103].

#### 3.2.3. Covalent Binding

Enzymes can also be covalently bonded to support matrices by water-insoluble linkers. Nucleophilic groups (amino groups, carboxyl groups, sulfhydryl groups, hydroxyl groups, phenolic groups and thiols) present in the amino acids of the enzyme, which are not involved in the active site, are used to form the covalent linkage. The reaction must be conducted under specific working conditions, as low temperature, neutral pH and low ionic strength, in order to avoid the loss of functional enzymes. This technique has proven successful in significantly improving the lifetime of biosensors [104].

#### 3.2.4. Polymeric Films

Polymeric films have also been used extensively to immobilize enzymes, as well as act as permselective layers. Functionalized polymers are used to trap enzymes by means of ion-pair interactions and hydrogen bonds, stabilize enzymes by shielding active sites from polar aqueous environments and diminishing the water activity around the protein. In order to enable analyte detection, the polymer selected must allow analyte and other co-factors to reach and diffuse out of the active site [105].

Biofouling and interferents are the two major problems affecting the performance of a biosensor, especially for implantable devices. In particular, electroactive substances have proven to be challenging when electrochemical detections are made on physiological complex matrices, such as blood [106]. Ideally, biocompatible membranes should act as a selective barrier, preventing interferents but not analyte from reaching the enzyme biosensor layer. 

Different types of membranes have been introduced in biosensor design, some examples are Nafion^®^ (Wilmington, DE, USA), cellulose acetate and polystyrene.

Nafion^®^, a perfluorinated sulfonated membrane, has been commonly used as a biocompatible membrane in biosensors. It is easy to cast, chemically inert, mechanically strong, thermally stable and resists fouling. Moreover, Nafion^®^ also produces a structure with hydrophilic channels within a hydrophobic matrix, and strong excludes anionic interferents [107]. Cellulose acetate deposition is able to reject electrochemically-active compounds that could affect electrochemical measurements in complex matrices such as blood serum [108].

Polystyrene is now being used for immobilization. It is non-toxic, chemically inert, rigid and adsorbs well to electrodes. It also shows excellent biocompatibility, high affinity and low molecules permeability [109].

The general definition of permeability (P) of molecules across a membrane can be expressed as:

(8) $$P=\frac{KD}{\Delta x}$$

where K is the partition coefficient, D is the diffusion coefficient, and Δx is the thickness of the membrane [110].

The ability of specific polymer membranes, usually called *inner membranes* (*IMs*) because of their localization in the deeper layers of the amperometric biosensor, to block the electroctive interfering molecules and to allow the passage of substrates or enzyme reaction by-products (H_2_O_2_), is measurable by means of specific parameters indicated as *IM permselectivity* (IMPS) and *IM permeability (IMP)*.

*IMPS* represents the percentage of interference on the detection of the studied analyte. For example, the permselectivity of a biosensor that measures H_2_O_2_ in the presence of ascorbic acid (AA) is expressed as follows:

(9) $$IMPS\left(\%\right)=\frac{IAA}{IHP}\times 100$$

were I_AA_ and I_HP_ represent the currents produced by the oxidation of AA and H_2_O_2_ respectively. Ideally, S% should be close to zero for biosensor applications [111].

*IMP* is used to quantify the capability of different polymeric films to allow the diffusion of the target analyte to the surrounding transducer [111]. *IMP* is expressed as follows:

(10) $$IMP\left(\mathrm{analyte}\right)\%=\frac{Ianalyte\;on\;polymer-covered\;transducer\;}{Ianalyte\;on\;bare\;transducer}\times 100$$

where I are the currents produced by the oxidation of the analyte. 

As evident in Equations (1) and (2), in first generation biosensors optimum analytical performance can be attained when the ratio of target analyte to O_2_ is <1 [112,113]. In some *in vivo* applications this ratio is not met as in the detection of extracellular glucose in subcutaneous tissue where glucose/O_2_ ratio is around 30 under physiological conditions. This results in biosensor saturation at higher glucose concentrations. In order to reduce the signal saturation, permselective *outer membranes* (*OM*) have been used in order to reduce the diffusion of the analyte without affecting the oxygen diffusion [112,113].

The permeability of the *outer membrane (OMP)* can be evaluated experimentally [112,113] and the apparent diffusion coefficient (Dp) of the analyte can be calculated by means of the following equation:

(11) $$N_{A}=\frac{Dp\;}{L}\left\{\left[CG\right]i-\;\left[CG\right]f\right\}$$

where N_A_ = Δm/A Δt is the flux over the area (A) of the OM, Δm is the mass of the analyte that diffuses in the time Δt, L is the thickness of the outer membrane and [CG]I and [CG]f are the concentrations of the analyte between the two sides of the membrane.

Polymeric films can be used to block interferents, such as ascorbic acid, the most concentrated electroactive compound in brain extracellular fluids [114,115,116], increasing the selectivity and specificity of biosensors. Non-conductive films obtained by the electropolymerisation of poly-phenylene-diamines (PPDs) are known to have excellent permselectivity and permeability properties allowing hydrogen peroxide produced by oxidase enzymes to freely diffuse while blocking oxidizable anions [111,116]. Other non-conducting polymers like polyphenols-based polymeric films [117] also show good permselectivity. In non-conducting polymers, it is possible to control the film thickness, by means of the applied potential or the electrochemical technique employed (for example, constant potential amperometry or cyclic voltammetry), generating a self-limiting process; the growth (thickness) of conductive polymers depends also on the polymerisation time and the amount of exchanged charges (electrons) during electro-synthesis.

Different kind of polymeric conducting films, such as polypyrrole [118,119,120] polyaniline [121,122] or PEDOT [123,124], are also used for enzyme immobilization. Conducting polymers have unusual electronic properties and have been proven to enhance speed, sensitivity and performances of biosensors [125]. They are obtained by means of several polymerisation techniques, such as electrochemical, galvanostatic or potentiostatic procedures. By modifying different parameters, it is possible to regulate polymer thickness and performance [126].

Molecularly imprinted polymers are also being used to develop reliable and stable biosensors for monitoring compounds, even under severe conditions [127]. Sites are created in polymer matrices for recognition and catalysis. In this approach, polymers are formed in the presence of a model molecule, and after polymerization, cavities with the desired shape and functional groups are present in the imprinted materials.

#### 3.2.5. Enzyme Stabilizers

Proteins are polyampholytic, so they are able to interact with adjacent polarized compound [89]. Hence, another common strategy to improve biosensor performance is the use of different types of enzyme stabilizers, such as polyelectrolytes, dextrans [128,129] and polyethyleneimmine [58,130]. The polycationic resin polyethyleneimine has been cast in several types of biosensors to stabilize enzymes, like glutamate oxidase [131,132] or lactate oxidase [133] by increasing enzyme stability through the formation of polyanionic/polycationic complexes and decreasing the electrostatic repulsion between the enzyme substrate and biosensor elements [132]. Glycerol has also been used to stabilize enzymes, increasing biosensor stability over time [134].

## 4. Biological Matrices: Composition and Matrix-Related Detection Problems

In biosensing, when the detecting device is directly in contact with biological fluids (Table 3), undesired signals from interferents are a serious and prevailing problem. There are a number of low and high molecular-weight interfering compounds, such as oxidizable acids (e.g., ascorbic acid, uric acid, homovanillic acid) and bases (e.g., oxidizable catecholamines and indolamines). Biological fluids may also contain drugs and their metabolites (e.g., acetominophen). Moreover, proteins (high molecular weight interferents) at high concentrations can adsorb nonspecifically to the transducer surface, interfering with detection of target molecules, which are often present at low concentrations. [135].

All biological matrices can contain compounds that cause biofouling or enzyme inactivation: principally, these kinds of molecules are low and high molecular weight proteins, but may also include water soluble small molecules (e.g., sugars) and hydrophobic compounds (e.g., lipids). All of these compound can cause electrode passivation and/or biofouling.

In order to face the issue of sensor impairment, several membrane coatings, such as Nafion, polyurethanes with phospholipid polar groups, 2-methacryloyloxyethyl phosphorylcholine, hyaluronic acid, humic acids, phosphorylcholine, polyvinyl alcohol hydrogels, have been used. These membranes reduce the impact of biological fluids on biosensors, minimizing protein adsorption while still allowing target analytes to reach the sensing surface. However, such strategies are less effective against low molecular weight protein fragments and large charged cell deposits [136].

Mono- and divalent cations are present at high concentrations in biological fluids, and can activate or inhibit many enzymes. These cations can act as allosteric effectors without participating in the enzymatic reaction, or alter the conformation needed for catalytic activity. The charge and the size of the ion present in the catalytic site of an enzyme represents one of the important issues that determines which metal ion is able to inhibit an enzyme [137]. In some cases, the interaction of monovalent cations with enzymes is important for catalysis; K^+^ and Na^+^, for example, stabilize glucose oxidase against thermal denaturation [138].

Electrode passivation can arise with non-specific adsorption of proteins and lipids. Deposition of polymeric films, such as *o*-phenylenediamine, polyeugenol, polypyrrole and other films (conducting or nonconducting) or specific membranes, as Nafion, polymethylcellulose or hydrogels, can prevent passivation, although often at the expense of response time. Carbonanotubes have also been used to minimize electrode fouling, for example, from oxidation of NADH [139,140,141].

### 4.1. Saliva

Saliva is a clear, slightly acidic exocrine secretion that contains oral bacteria and food debris. It is composed of several compounds, including cations, such as sodium, potassium, calcium, magnesium, and anions, such as bicarbonate, and phosphates. Saliva also contains nitrogenous compounds, such as urea and ammonia, as well as immunoglobulins, proteins, enzymes and mucins [142]. Saliva glucose concentrations range approximately from 20 to 200 µmol/L in normal and diabetic patients, closely follow circadian blood glucose fluctuations [143]. Normal saliva is a complex solution derived from parotid, submandibular, sublingual, and minor gland secretions, and may also include bacteria, leukocytes, epithelial cells, and gingival crevicular fluid, that make the measurement of components of saliva difficult [144]. Concentrations of alcohol, glucose and lactate measured in saliva correlate well with concentrations in blood serum [145]. Saliva also contains enzymes, such as amylase [145], which transforms complex sugars into simple ones; thus, its activity could interfere with the accurate detection of some analytes, such as glucose. Normal saliva pH ranges from 6.5 to 7.4 and is related to saliva buffers (bicarbonate) and oral hygiene.

### 4.2. Urine

Na^+^, K^+^, Ca^2+^, Mg^2+^ and NH_4_^+^ account for nearly all the cations present in urine, while chloride, sulphate, phosphate and bicarbonate account for about 80% of the anions present [146]. Cells and proteins are negligible. Urine pH normally ranges between 4.6 and 8, and is related to diet and overall health.

### 4.3. Blood, Plasma and Serum

Blood is a complex mixture of plasma (the liquid component), white blood cells, red blood cells, and platelets. Plasma is composed of 90% water and represents about 55% of blood volume. Serum is blood plasma without fibrinogen. In serum and plasma, several water soluble compounds are present, such as nutrients, hormones and electrolytes. It is also possible to find drugs and proteins, such as globulins (including antibodies), fibrinogen (blood clotting factor), albumin (major protein constituent) and other clotting factors [147]. There may be up to 10,000 proteins in serum, including immunoglobulins, albumin, lipoproteins, haptoglobin, and transferrin. Blood is a highly buffered fluid, with normal blood pH ranging from 7.35 to 7.45.

### 4.4. Extracellular Fluid (ECF) and Brain Extracellular Fluid (bECF)

ECF is mainly composed by ions (Na^+^, Cl^−^ and Ca^2^^+^), glucose, amino acids and ATP, with negligible protein content. One of the major applications of amperometric biosensors involves their implantation in specific brain regions, in direct contact with brain extracellular fluid (bECF). *In vivo* microdialysis allowed a deep characterisation of bECF composition. In particular, the ionic composition of bECF is well known (NaCl 147 mM, KCl 2.7 mM, CaCl_2_ 1.2 mM, MgCl_2_ 0.85 mM) [148]. Electroactive molecules are also present, such as ascorbic acid (AA). AA performs various functions at the neuronal level and is considered to be one of the most powerful antioxidants. Also, catecholamines, such as dopamine, noradrenaline and their major metabolites, such as 3,4-dihydroxyphenylacetic acid, 3-methoxytyramine and homovanillic acid (HVA), are present. In bECF, uric acid produced by the catabolism of purines [149,150] may be detected, as well as 5-hydroxy-tryptamine (serotonin) and its metabolite 5-hydroxyindoleacetic. bECF contains also many other molecules which can be detected by means of amperometric biosensors, such as glucose, lactate, glutamate, acetylcholine and choline [151].

### 4.5. Tears

Tears are produced by the lachrymal glands and can be used as an interesting fluid for non-invasive monitoring [152]. The amount of protein present in tears is negligible. The normal pH range is between 6.5 and 7.6.

### 4.6. Sweat

Sweat includes urea, uric acid, sugar, lactic acid, amino acids, and ammonia [153]; concentrations vary widely from person to person. The normal pH of sweat is around 5.5.

### 4.7. Changes in Biological Fluids Composition Related to Physiological and Pathological Conditions

Composition of the biological fluids described above may change in response to various physiological and pathological conditions. The pH of saliva and urine, for example, is closely related to diet; saliva pH can drop immediately after food consumption and low pH is related to poor oral hygiene. Some pathological conditions, such as inflammation, neurodegenerative diseases, infections or cancer, can also modify some fluid parameters, such as dissolved oxygen percentage, chemical composition and pH. These variations in composition of biological fluids can impact biosensor performance. For example, ischemia would decrease the response of first generation biosensors by virtue of their oxygen dependence (Figure 1) and over-production of Reactive Oxygen Species (ROS) and proteases could damage biosensors. Such variations in biological fluid composition are known. For example, a PET study confirmed ROS increase with striatal oxidative stress in patients affected by Parkinson’s disease [154]. Similarly, cancer, inflammation and infectious diseases, result in increased ROS production [155,156]. Notably, ROS are primarily responsible for biosensor ageing and enzyme-related loss of sensitivity due to the inactivation of catalytic sites or cofactors (reduction of apparent V_MAX_) or the oxidation of non-catalytic sites with consequent molecular rearrangement and reduced substrate affinity (increase of apparent K_M_). Neurodegeneration, inflammation and cancer are also responsible for the increase in proteases [156,157,158,159,160] that may damage biosensors.

## 5. Conclusions

Amperometric enzyme-based biosensors are complex analytical devices combining interdisciplinary knowledge, based on electrochemistry, materials science, polymer synthesis, enzymology and biological chemistry. Although biosensors can be fully characterized in a controlled laboratory environment, use in biological matrices, such as biological fluids, is more challenging. This latter step shows the difference between the research on biosensor (development) and the use of biosensor for research, as diagnosis and follow-up analytical devices.

## Figures and Tables

**Table 1:** Examples of oxidase enzymes used in amperometric biosensors.

Enzyme	Source	Substrate	References
Glucose oxidase	*Aspergillus niger*, E.C. 1.1.3.4	β-d-Glucose	[25,26,27,28]
Glutamate oxidase	*Streptomyces sp* EC 1.4.3.11	l-Glutamate	[24,29,30,31]
Alcohol oxidase	*Pichia pastoris* *Hansenula polymorpha* EC 1.1.3.13	Ethanol	[32,33,34,35]
Lactate oxidase	*Pediococcus sp*. *Aerococcus viridians* EC 1.1.3.2	l-Lactate	[28,36,37,38]
Ascorbate oxidase	*Cucurbita sp* EC 1.10.3.3	l-Ascorbic acid	[39,40]
Cholesterol oxidase	*Streptomyces sp* porcine pancreas EC 1.1.3.6	Cholesterol	[41,42,43]
Choline Oxidase	*Alcaligenes sp* (EC 1.1.3.17)	Choline Acetylcholine	[44,45]
Laccase	*Trametes pubescens* *Paraconiothyrium variable* *Trametes versicolor* (EC 1.1.3.4)	Polyphenols	[46,47,48]
Tyrosinase	Mushroom EC 1.14.18.1	Monophenols Dihydroxyphenols Bisphenol A	[49,50,51]

**Table 2:** Examples of dehydrogenase enzymes used in amperometric biosensors.

Enzyme	Source	Substrate	References
Alcohol dehydrogenase	*Saccharomyces cerevisiae* E.C. 1.1.1.1	Etanol	[54,55,56,57]
Glutamate dehydrogenase	bovine liver E.C. 1.4.1.2	l-Glutamate	[58,59,60]
Glucose dehydrogenase	*Pseudomonas sp.* *Escherichia coli* EC 1.1.1.47	Glucose	[61,62,63,64]
Lactate dehydrogenase	Rabbit muscle Chicken heart EC 1.1.1.27	l-Lactate	[62,65,66]

**Table 3:** Qualitative composition of selected biological fluids presented in this review.

Fluid	Cations	Anions	Proteins	Metabolites	Nutrients
Saliva	++	+++	++	−−−	−−−
Urine	++	+++	−−	+++	−−−−
Blood	++	++	+++	+++	+++
ECF	++++	+++++	−−	++	+
Tears	++	++	−−	+	+
Sweat	+++	+++	−−	+	+
